# Intensive insulin therapy-induced severe hypoglycemia does not affect long-term functional and cognitive outcome or health-related quality of life

**DOI:** 10.1186/cc9819

**Published:** 2011-03-11

**Authors:** RE Harmsen, JG Hofhuis, J Korevaar, F Van Braam Houckgeest, JP Van der Sluijs, MJ Schultz, PE Spronk

**Affiliations:** 1 Academic Medical Center, Amsterdam, the Netherlands; 2 Gelre Hospitals, Apeldoorn, the Netherlands; 3 Tergooi Hospitals, Hilversum, the Netherlands; 4 Medical Center Haaglanden, The Hague, the Netherlands

## Introduction

Intensive insulin therapy (IIT) has been shown to reduce mortality in intensive care patients [1,2]. A large study on IIT was stopped prematurely due to high incidence of severe hypoglycemia (SH) (blood glucose level (BGL) <40 mg/dl) [3]. It remains unclear, however, whether short episodes of IIT-associated SH are truly harmful [4]. We investigated long-term quality of life and functional and cognitive outcome in patients with and without IIT-associated SH.

## Methods

Three hospitals developed and implemented an evidence-based guideline for IIT; we collected all BGL measurements and patient demographics for the 2 years after implementation. We captured all patients with SH, and randomly selected the same number of patients without SH as controls. To evaluate long-term outcome, we used the following scores: Glasgow Outcome Scale (GOS), Short-Form (SF)-12 for health-related quality of life (HRQOL) expressed as physical (PCS-12) and mental component score (MCS-12), Informant Questionnaire on Cognitive Decline in the Elderly (IQ-CODE) and the Modified Blessed Dementia Rating Scale (MBDRS) by proxies.

## Results

Our analysis included 93 patients, 43 patients with at least one episode of SH, and 50 control patients. Median length of an SH episode, assuming linear changes of glucose values between measurements, was 20 (10 to 50) minutes. Patient demographics (age, gender, APACHE II scores) were similar. Median length of ICU stay was longer in patients with SH, 12 (6 to 20) versus 4 (8 to 23) days (*P *< 0.001). Median BGL was lower in patients with SH, 101 (97 to 106) versus 113 (102 to 123) mg/dl (*P *< 0.001). Outcome indicators were similar between patients with at least one episode of SH and control patients: GOS; 1 (1 to 1) versus 1 (1 to 2) (*P *= 0.173); PCS-12; 44 (33 to 50) versus 42 (34 to 52) (*P *= 1.000); MCS-12; 49 (38 to 56) versus 45 (35 to 52) (*P *= 0.093), IQ-CODE; 3.0 (3.0 to 3.3) versus 3.0 (3.0 to 3.1) (*P *= 0.116) and MBDRS 1; (0.5 to 1.5) versus 0.5 (0.5 to 2.5, *P *= 0.734).

## Conclusions

Neither long-term functional and cognitive outcome, nor HRQOL of patients who encountered IIT-associated SH differed from patients who never had SH. However, it should be noted that the analyzed groups are small. Our data suggest IIT-associated SH not being harmful.
